# Challenges and adaptations in implementing an English-medium medical program:a case study in China

**DOI:** 10.1186/s12909-018-1452-3

**Published:** 2019-01-09

**Authors:** Miao Yang, Patricia S. O’Sullivan, David M. Irby, Zexin Chen, Chun Lin, Changmin Lin

**Affiliations:** 1 Department of Foreign Languages, Shantou University Medical College, Shantou, China; 2 Center for Faculty Educators, School of Medicine, University of California San Francisco, San Francisco, USA; 3 Department of Histology & Embryology, Medical Education Assessment and Research Center, Shantou University Medical College, Shantou, China

**Keywords:** English as the medium of instruction, Challenges, Adaptive teaching and learning strategies

## Abstract

**Background:**

Increasingly, non-English speaking countries use English as the medium of instruction (EMI) to teach academic subjects. This study investigated the challenges and adaptation strategies of teachers and students in an EMI medical education program in China.

**Methods:**

Data were collected on EMI and non-EMI students’ test performances and student and teacher perceptions of the program. Test scores and survey results were analyzed using SPSS. Focus group transcripts and open-ended comments from surveys were examined using thematic coding.

**Results:**

There were no significant differences in admission and graduation test scores for EMI and non-EMI students. Four challenges with the EMI program were identified: (1) insufficient/inappropriate teaching materials, (2) unsatisfactory teaching, (3) inadequate class interactions, and (4) failure to teach medical humanities. To address these challenges, teachers and students used adaptive strategies, such as the use of alternative textbooks, self-learning skills and Chinese language.

**Conclusions:**

EMI programs are difficult to initiate, requiring faculty development and institutional support, and student self and peer group learning strategies to be successful. The adaptive strategies employed by both students and teachers offer insights into how other EMI programs might strengthen their implementation.

## Background

In response to globalization, English has emerged as the international language of academic communication in the last several decades. This has led to a global phenomenon of using English as the medium of instruction (EMI) to teach academic subjects in non-English speaking countries [1–3]. This is particularly true in the field of medical education where the majority of published biomedical science research and professional information is in English [4]. Medical education is now a global enterprise involving increasing collaboration between medical schools in different countries [5] as a result of the globalization of healthcare delivery [6]. However, there is a paucity of research on the effectiveness of EMI medical programs and the challenges of implementing such programs.

Early research on EMI, particularly outside of medicine, tended to focus on the short-term and classroom-based learning gains from the EMI programs. These studies focused either on English abilities [7, 8] or on the acquisition of subject matter knowledge [9]. Gradually, the scope of this research has broadened to consider how EMI programs are culturally and socially embedded in non-English speaking cultures and how they impact the cognitive and affective perspectives of the stakeholders. Some studies offered insights into local EMI practices, and address the socio-political context unique to each EMI program [3, 10, 11]. These studies examined the co-existence of local and global culture in institutional contexts, attending to the tension, synergy, and negotiation between the two cultures, and identifying effective pedagogical strategies and practices [3].

However, EMI medical education research has not followed this trend toward examining the cultural and social aspects of EMI programs. Among the few studies of EMI medical programs, most investigations were of a short duration of learning or of a single course [12–14]. For example, Joe and Lee [12] investigated the influence of EMI on the degree of lecture comprehension for the students based on three class hours (150 min totally) in a medical course, which might not be sufficient time to observe significant effects.

The challenge associated with EMI programs in medical education derives from the greater complexities and locales of teaching and learning. In medical education, students learn both basic science and clinical content in the context of classroom and clinical settings (where advanced preparation is not always possible). Instruction in subjects such as professionalism, communication, ethics and interprofessional teamwork requires active use of language, concepts and practice by the learner [15]. As a result, instructional strategies that actively engage teachers and learners in dialogue are being used increasingly [16] . However, such dialogue carries with it cultural norms and values, which is easier to communicate in one’s native language [17, 18]. This creates increased challenges to learning in EMI programs.

The current research was conducted in a Chinese context where the EMI medical program is 10 years old. It addressed the following research questions:

1. Do EMI and non-EMI medical students perform equivalently?
2. What are the challenges of implementing an EMI program?
3. How do teachers and students adapt to meet these challenges?

## Methods

### Research setting

The setting for this study was Shantou University medical college (SUMC), China. Founded in 1983, SUMC mainly enrolls high school graduates for the seven-year clinical medicine curriculum. The students take an internship in the fourth year and conduct postgraduate study in the last two years. In 2007, SUMC established an EMI program to produce clinicians who are internationally competitive and highly qualified in medical service. SUMC selects EMI students based on their performance in: (1) the college-based English courses that teach skills of learning, thinking and researching; (2) a written English test similar to the International English Language Testing System (IELTS); and (3) an English interview that tests oral English, communication abilities and critical thinking skills. The mean score of the participant students in College English Test Band-4 (a compulsory English proficiency test for all non-English major undergraduates in China) is about 600 (full score = 710), which means the students’ level is higher than 90% of the norm population [19]. Each year, 30 EMI students (about 17% of the total) enroll at the end of the first semester. The EMI scholarship committee eliminate students with poor academic performance annually. Some students may quit because of personal reasons. As a result, there were 203 EMI students (enrolled in 2007–2016) at the time of this research. After the first four-year study (including general courses such as English, Philosophy, Physics, and Chemistry, and basic medical science courses such as Biochemistry, Molecular Biology, Cytobiology, Anatomy, Histology and Embryology), they volunteer to take United States Medical Licensing Examination (USMLE) Step-1. The passers undergo 8-week preparation for internship training in U.S., Canada, England and the Netherlands. After taking intensive training at a language university, the EMI teachers receive accreditation from a committee composed of English-speaking medical professors and English teachers. They are required to deliver a lecture in English and answer questions from the committee to display their pedagogical skills and communication skills in English.

### Participants

The research participants comprised three groups: students receiving EMI instruction from 2007 to 2016 (*N* = 188), EMI teachers from a range of medical disciplines (*N* = 74), and faculty administrators who have been directly involved in the development and implementation of the EMI program (*N* = 3).

### Data collection

This study adopted a case-study approach combining different methods to illuminate the 10-year EMI program from various angles, with the recognition that every EMI context is unique and dynamic. The SUMC’s Research Ethics Committee approved the study. Data were collected from students’ test scores, survey responses and focus group discussions. Teachers completed surveys and focus groups.

#### Test scores

In the past ten years, five classes of students (Class 2007–2011) have taken the Chinese Medical Practitioner Examination (CMPE) in their sixth year of study. Only the sub-tests scores of two classes (Class 2009 and 2010, which took the examination in 2015 and 2016 respectively) were available for analysis due to the information disclosure policy in China. The researchers used these scores as graduation test scores to check differences in learning outcomes between EMI and non-EMI students. The Chinese College Entrance Examination scores of these two classes served as the benchmark of the students’ general capacities before entering the EMI program. In addition, the students’ USMLE scores represented an international standard of the EMI learning outcomes.

#### Questionnaire survey

First, the researchers conducted semi-structured interviews to develop a comprehensive understanding of the EMI program and identify important issues to include in the questionnaire. Five EMI students, four EMI teachers, and three administrators were invited to the interviews. Results from the interviews informed the items to be included on the questionnaire.

Besides questions that asked about grades, years of teaching/learning experience, and general comments on EMI, there were 51 items (teacher’s version) and 44 items (student’s version) that were rated on a 6-point Likert scale ranging from “Strongly disagree” (1) to “strongly agree” (6). The questionnaire had both English and Chinese versions. The Chinese version was distributed to the participants for the sake of better understanding.

#### Focus group

Three focus groups were organized using the focus group guide created by Tiberius [20]. One focus group included 3 EMI teachers, and two separate focus groups included 2 and 3 students respectively. These participants were a different group from the interview participants mentioned above. They were selected using a maximum variation sampling method [21] to ensure both the diversity and commonality in EMI-related views and practices. For better communication of ideas, the focus groups were conducted in Chinese.

### Data analysis

Quantitative data collected from the test scores and the questionnaire surveys were analyzed using the Statistical Package for Social Science (19.0). First, descriptive analysis provided frequencies and percentages. Paired t-test analyses (Welch’s t-test were used to remedy the unequal sample sizes between EMI and non-EMI students) were then conducted to identify possible discrepancies in test performance between EMI and non-EMI students. All qualitative data from the narrative comments in the survey and focus group discussions underwent three stages of data transformation (description, analysis and interpretation), using a constant comparative method of analysis [22, 23]. Textual data cited in this paper was translated by an experienced English teacher at SUMC.

## Results

The results are presented in the order of the three research questions and the sources of data. The first question was: Do EMI and non-EMI medical students perform equivalently?

### Comparison of EMI and non-EMI students’ test score

At entry to the program, EMI and non-EMI students’ admissions scores (Chinese College Entrance Examination) were equivalent as measured by a paired t-test. In addition, their graduation test performance showed no significant differences using a paired t-test (*p* > 0.05). On the Chinese Medical Practitioner Examination scores, there were no significant difference overall, but sub-scores for some subject matter areas differed (Table 1). There is a small to medium effect size favoring the EMI learners. For performance on USMLE scores, SUMC EMI students were compared with non US/Canadian students. From 2012 to 2017, the pass rates of SUMC students (first time takers) were 100, 96, 96, 96, 80, 88% respectively, while those of the non US/Canadian students were 76, 79, 78, 78, 78, 78% [24].

**Table 1:** Comparison of CMPE Scores between EMI and Non-EMI Students

Subject	2015						2016					
	Non-EMI (*N* = 226)		EMI (*N* = 30)		Sig.	*d*_Cohen_	Non-EMI (*N* = 222)		EMI (*N* = 25)		Sig.	*d*_Cohen_
	M	SD	M	SD			M	SD	M	SD		
Preventive	21.38	2.98	23.07	2.15	.003^a^	.583	19.77	2.92	20.80	2.83	.095	.354
Basic	53.15	8.00	55.47	7.10	.132	.294	56.64	6.96	60.80	6.58	.005^a^	.601
Humanistic	27.75	3.60	29.27	2.84	.028^a^	.432	28.41	2.84	28.40	2.77	.981	−.004
Clinical	325.53	34.60	335.7	22.92	.119	.304	332.45	31.89	343.32	30.54	.106	.342
Remembering	83.55	10.46	87.80	7.84	.033^a^	.417	70.81	7.17	73.64	6.18	.059	.400
Understanding	128.43	13.69	134.53	9.07	.019^a^	.461	101.76	10.71	105.92	12.00	.070	.384
Applying	215.82	21.60	221.17	15.58	.191	.255	264.72	23.78	273.76	22.57	.071	.382
Total^b^	427.81	43.74	443.5	30.08	.058	.370	437.28	39.22	453.32	38.38	.057	.410

^a^Significant level ≦ 0 .05

^b^Total score = 600; Passing line = 360

### Comparison of students’ and teachers’ views on EMI learning/teaching

The second research question was: What are the challenges of implementing an EMI program? Findings from the questionnaire survey describe how students and teachers evaluated the EMI teaching and learning and what challenges emerged.

Overall, 188 questionnaires were collected from 203 EMI students (response rate = 92.6%) and 74 from 100 EMI teachers (response rate = 74%). Both the EMI students and teachers described the superiority of English over Chinese textbooks, reporting that the English ones appear to be more updated, practical, integrated (i.e. emphasizing the combination of basic science knowledge and clinical practice), and focused (Table 2). But the students were slightly negative about their own efficiency in using English textbooks to study (Mean = 3.968), while the teachers perceived English textbooks as more efficient for learning (Mean = 4.676; *P* < .001).

**Table 2:** Comparison of Students’ and Teachers’ Views on EMI Teaching/Learning

Items		Students		Teaachers			
		Mean	SD	Mean	SD	Sig.	d_Cohen_
Superiority of English textbooks	Updated	5.048	.953	4.757	1.168	.059	−.286
	Practical	4.534	1.039	4.514	1.162	.887	−.019
	Integrated	4.767	1.061	4.576	.994	.164	−.183
	Focused	4.360	1.262	4.311	1.072	.752	−.040
	Efficient	3.968	1.207	4.676	1.112	.000^a^	.599
Teaching effectiveness	Not clear	4.074	1.123	3.695	1.417	.064	−.316
	Not substantial	3.878	1.199	3.559	1.489	.137	−.251
	Translating PowerPoints	4.307	1.107	2.729	1.412	.000^a^	− 1.332
Medical humanities	Respect	5.063	.741	4.529	.615	.000^a^	−.738
	Medical ethics	4.984	.768	4.529	.706	.002^a^	−.599
	Communication skills	4.820	.863	4.294	.836	.001^a^	−.612
English proficiency	GE skills	4.757	1.018	4.892	.973	.328	.134
	Academic speaking	4.799	.979	4.973	.860	.182	.184
	Academic reading	5.185	.930	5.135	.816	.685	−.056
	Academic writing	4.937	.954	4.946	.858	.941	.010
Self-learning	Identifying problems	4.413	.945	4.324	.742	.424	.100
	Raising questions	4.180	1.171	4.324	.923	.342	.130
	Seeking for answers	4.862	.947	4.392	.791	.000^a^	−.519
	Independent thinking	4.683	.908	4.405	1.019	.033	−.296

Full scale = 6 (strongly agree)

^a^Significant level ≦ 0 .003 to correct for Type I error of multiple tests

Both the teachers and the students were not satisfied with the effectiveness of classroom instruction, reporting that teachers did not deliver the subject knowledge clearly and in enough depth because of EMI. However, their views differed on whether some teachers simply translated the PowerPoints from Chinese into English (*P* < .001).

Half of the teachers thought EMI class was lively, while the other did not. This was attributed to educational concepts, personal styles and English language proficiency of the teachers. About half of the teachers admitted that they had to use Chinese to guide interactions. Possibly, the students’ lack of medical knowledge may have contributed to poor interaction. 78% of the students thought class interaction had improved by their senior level after they had mastered more medical knowledge.

Both the teachers and the students positively evaluated medical humanities (MH) learning as reflected in the students’ clinical performance when 1) showing respect for patients’ privacy, rights and interests; 2) performing with proficient communication skills when asking patients questions and performing physical examinations; and 3) applying medical ethics and legal knowledge to the analysis of clinical cases. The students appeared to have a higher evaluation of their own performance than the teachers had.

Both the teachers and the students thought highly of the EMI program in improving English language skills for both general and academic purposes. Cognitively, they also believed that the program had helped to cultivate the students’ independent thinking skills and self-learning skills. The motivation of self-learning was attributed to the unsatisfactory EMI teaching effectiveness by 97% of the students, who claimed that some EMI classes had taught less than expected and they had to make it up after class by themselves. Meanwhile, 86% of the students said the difficulty of learning medicine in English required more effort to study outside the class.

### Adaptations to challenges

The third research question was: How do teachers and students adapt to meet these challenges? Content analysis of focus groups data not only identified some of the challenges that are generally consistent with questionnaire data, but also provided evidence of the teachers’ and students’ practices in addressing these challenges. They are summarized in Table 3 and explained briefly here.

**Table 3:** Adaptation to Challenges in EMI Program

Challenges	Students’ practice	Teachers’ practice
A. Insufficient or inappropriate EMI teaching resources	* Using the English textbooks selectively based on their own learning capabilities. Some stick to original English textbooks; some manage to find simplified versions; most of them use both English and Chinese textbooks simultaneously.	* Using the English textbooks selectively based on their understanding of the students’ learning needs, usually with a combination of Chinese textbooks and updated English textbooks. * Encouraging students to use Chinese textbooks
B. Unsatisfactory teaching efforts	* Previewing before the lectures; * Learning autonomously after class to make up the content not taught/understood clearly in class; * Searching learning materials from various sources, such as online medical forum and database (e.g. UpToDate); * Turning to study groups and peers for help	* Devoting a great deal of time preparing for lessons * Improving organization of teaching content by setting up clear learning objectives and structuring the content more logically; * Writing scripts to improve fluency; * Revising PPTs to improve teaching quality year after year
C. Inadequate classroom interaction	* Asking questions after class (in Chinese); * Participating in PBL discussions	* Answering questions after class (in Chinese); * Plan for interactive sections in advance (only practiced by a few teachers); * Interacting with students during PBL sections (but not all EMI teachers are involved in PBL)
D. Failure to teach medical humanities issues	* Learning and discuss MH issues in PBL discussions; * Learning MH from other courses; * Participating in pre-internship and volunteer activities	* Including case analysis in class instruction when MH issues might be mentioned (but not very often) * Involving students in MH discussions in PBL

To adapt to the English textbooks, both the teachers and the students used Chinese textbooks simultaneously for lesson planning and study. They displayed high flexibility in combining various sources to meet different needs.

To make up for the unsatisfactory quality of teaching, the students did considerable self-learning, including previews and reviews using online learning resources. They also relied on peer support after class. Some teachers would put more efforts to lesson preparation, revising PowerPoints to be more focused and logical than would other teachers.

Not comfortable with in-class interaction in English, some students liked to ask questions after class in Chinese, when the teachers responded in Chinese. In addition, Problem-based Learning (PBL) discussions helped to improve the interaction because both the teachers and students could prepare in both language and subject knowledge. The students and the teachers acknowledged the benefits of including MH issues into PBL discussions. The students also attributed MH learning to some Chinese speaking courses.

## Discussion

Overall, this study identified challenges involved in EMI program at SUMC. While the EMI students performed well in their examinations, they found instruction in English to be challenging. The challenges are related to teacher skill in English and with the ability to address cultural issues in English.

In this study, EMI and non-EMI students were comparable at entry and graduation in terms of academic performance. This finding is not exactly in line with other studies. There is a common worry that EMI instruction may compromise subject content. Yip et al. [25] found that the EMI students in Hong Kong secondary schools performed less satisfactorily in science learning than their non-EMI counterparts. In Amman, Al-Sebaee [26] found that the students saved more time and effort but had higher comprehension ability of medical knowledge if they used Arabic instead of English as the medium of instruction. While EMI students in our study also identified challenges, the short-term negative effects were mitigated by students’ progress in both English proficiency and subject matter knowledge.

We identified challenges reflecting how EMI teaching and learning are deeply affected by the social and cultural factors in the local context as well as the limited English proficiency of teachers and learners. Despite these challenges, the students and teachers at SUMC created adaptive strategies to improve teaching and learning including use of other resources.

Others [2, 27] have recognized the problems of insufficient teaching resources due to insufficient investment in education and the difficulty of obtaining online resources in some developing countries. At SUMC, teachers and students had English textbooks purchased for them. Later, the college bought access to ClinicalKey Index for all teachers and students. Yet, some clinical teachers complained that they lacked English textbooks that were specialty specific. They might have felt obligated to teach everything even more than in Chinese in order to feel confident that they had covered all topics. With limited English language proficiency, the teachers found it difficult to simplify concepts and clarify complex topics in English, a phenomena noted by others [27]. Therefore, they appeared to be more textbook-dependent than they were in Chinese teaching.

The EMI students actively participated in PBL using English textbooks. They also kept the Chinese textbooks to structure a bilingual lexicon and build up awareness of clinical cases specific to China. The use of textbooks in one’s primary language occurs in other EMI contexts as well [27]. What is uniquely challenging for EMI medical study, is the need to use imported textbooks for local needs of learning and clinical practice. This process of appropriation involves adopting the pedagogical tools available for use in particular social environments and then internalizing the ways of thinking and learning [28].

The anxiety associated with expressing one’s self spontaneously in English prevented teachers and students from active interaction in class, confirming the finding that EMI teachers tend to adopt a more didactic approach and use less interactive activities than non-EMI teachers [25]. It is more demanding for teachers to communicate abstract, complex concepts accurately in a foreign language. While a teacher can handle a lecture after good planning, a discussion is more challenging since it cannot be planned in advance.

The students and the teachers adopted the pragmatic approach of using primary language to remedy the inadequate classroom interaction, as commonly reported in EMI research [28–31]. Some researchers consider this bilingual practice to be an important coping strategy that helps EMI learners to negotiate meaning of unfamiliar terms and difficult content [31].

PBL discussion is another solution to improving classroom interaction. This finding corresponds with the notion that the EMI innovation is not simply an English version of the indigenous program, but “a lever for forcing change in higher education pedagogy” [2]. Although PBL is not an essential element in EMI medical programs, the combination of PBL with EMI at SUMC helped to introduce advanced educational concepts and subsequently bring about changes in teaching and learning behaviors.

Faculty support and development is also important to improve classroom interaction. EMI teachers need to develop pedagogic content knowledge (PCK), i.e. the ability to transform content knowledge into concepts that are accessible and understandable to learners at their level of development [32, 33]. With PCK, EMI teachers will be able to enter into dialogue with their students and help them develop a deeper understanding of content [25]. Training facilitating the teachers’ PCK and learning resources knowledge should help the teachers to adjust to EMI teaching pedagogically and cognitively. Additionally, student training is of the same importance to help them sort through various learning sources and develop active and independent habits of learning and thinking.

Medical humanities is an essential part of any medical curriculum [15]. Yet, it is an area that falls short both due to a lack of time that is spent covering content in English, but also the inability to express these concepts in English. Occasionally, some EMI teachers integrated MH into subject content and the students addressed MH issues in PBL discussions. Meanwhile, the EMI students took Chinese MH courses and participated in service-learning through volunteer activities. They applied MH learning from the Chinese-based program to PBL discussions and clinical practice. This explains why both the teachers and the students evaluated MH learning positively. It also illustrates the importance of institutional context and resources for a successful EMI program. An EMI program is context-specific. Social and Cultural resources other than language are also essential for learners to develop international visions and universal values.

## Limitations

This study was conducted in a natural setting, where institutional factors, such as admission policies, curriculum design and support, and instructional practices, have limited the ability to conduct an experimental study for a more systematic comparison of EMI and non-EMI learning. We chose instead to use a case comparative study to describe differences between the EMI and non-EMI instructional and learning processes. Thus, this study is embedded in the life of a medical school in modern day China.

## Conclusion

In this study, we examined an EMI medical school program in China in order to identify the challenges and successful local practices. While most of the challenges are similar to the other EMI programs, the adaptive strategies in this specific program may be instructive for other EMI programs.

The global policy of implementing EMI inevitably involves cognitive and conceptual changes and gives rise to the local norms of adjustment and appropriation. EMI programs are difficult to initiate, requiring faculty development and institutional support, and student self and peer group learning strategies to be successful. The adaptive strategies employed by both students and teachers at SUMC offer insights into how other EMI programs might strengthen their implementation of English programs.

## Abbreviations

CMPE: Chinese Medical Practitioner Examination
EMI: English as Medium of Instruction
IELTS: International English Language Testing System
MH: Medical Humanities
PBL: Problem-based Learning
PCK: Pedagogic Content Knowledge
SUMC: Shantou University Medical College
USMLE: United States Medical Licensing Examination
